# Influence of DNA characteristics on cell membrane damage stimulated by electrical short-circuiting via a low-conductive aqueous droplet in dielectric oil

**DOI:** 10.1371/journal.pone.0285444

**Published:** 2023-05-05

**Authors:** Yoshino Tsurusaki, Yuki Watanabe, Rika Numano, Takayuki Shibata, Hirofumi Kurita

**Affiliations:** 1 Department of Applied Chemistry and Life Science, Toyohashi University of Technology, Toyohashi, Aichi, Japan; 2 The Electronics-Inspired Interdisciplinary Research Institute, Toyohashi University of Technology, Toyohashi, Aichi, Japan; 3 Department of Mechanical Engineering, Toyohashi University of Technology, Toyohashi, Aichi, Japan; Khalifa University of Science and Technology, UNITED ARAB EMIRATES

## Abstract

We investigated gene electrotransfer using electrical short-circuiting via a cell suspension droplet in dielectric oil. An aqueous droplet of a few microliters placed between a pair of electrodes can be deformed by an intense DC electric field depending on the electric field intensity. When a droplet containing suspended cells and plasmid DNA elongates during deformation and connects the electrodes, the resulting short circuit can cause successful gene electrotransfection into various mammalian cells. We also investigated the influence of the electroporation medium on membrane permeabilization and the mechanisms of gene electrotransfection using short-circuiting via an aqueous droplet. One aim of this study was to investigate the influence of the conductivity of electroporation medium on gene electrotransfer stimulated by short-circuiting. It was found that low-conductivity medium with plasmid DNA resulted in a significant decrease in cell viability compared to the high-conductivity medium with plasmid DNA. Therefore, we demonstrated the influence of exogenous DNA on membrane damage stimulated by droplet electroporation using a low-conductivity medium. Thus, electrical stimulation with the combination of plasmid DNA and the low-conductivity medium resulted in tremendous membrane damage. Linearized plasmid DNA stimulated more significant membrane damage than circular DNA. However, the size of linear DNA did not influence the efflux of small intracellular molecules.

## Introduction

The application of intense electric pulses to cells transiently increases cell membrane permeability. This phenomenon is known as electroporation or electropermeabilization. Electroporation has been developed as a physical method to deliver cell-impermeable biomacromolecules into cells [1–3]. Gene transfection in vitro and in vivo is the most popular application [4, 5]. Recently, gene electrotransfer has been used for generating induced pluripotent stem (iPS) cells [6], genome editing [7], and gene therapy [8]. In addition, electroporation is also being developed for electrochemotherapy, where anticancer drugs are delivered into tumors [9, 10]. Irreversible electroporation (IRE), a nonthermal ablative technique, is an emerging cancer treatment [3]. In food processing industries, IRE is used in the extraction of valuable molecules from cells and sterilization [11].

**Fig 1:**
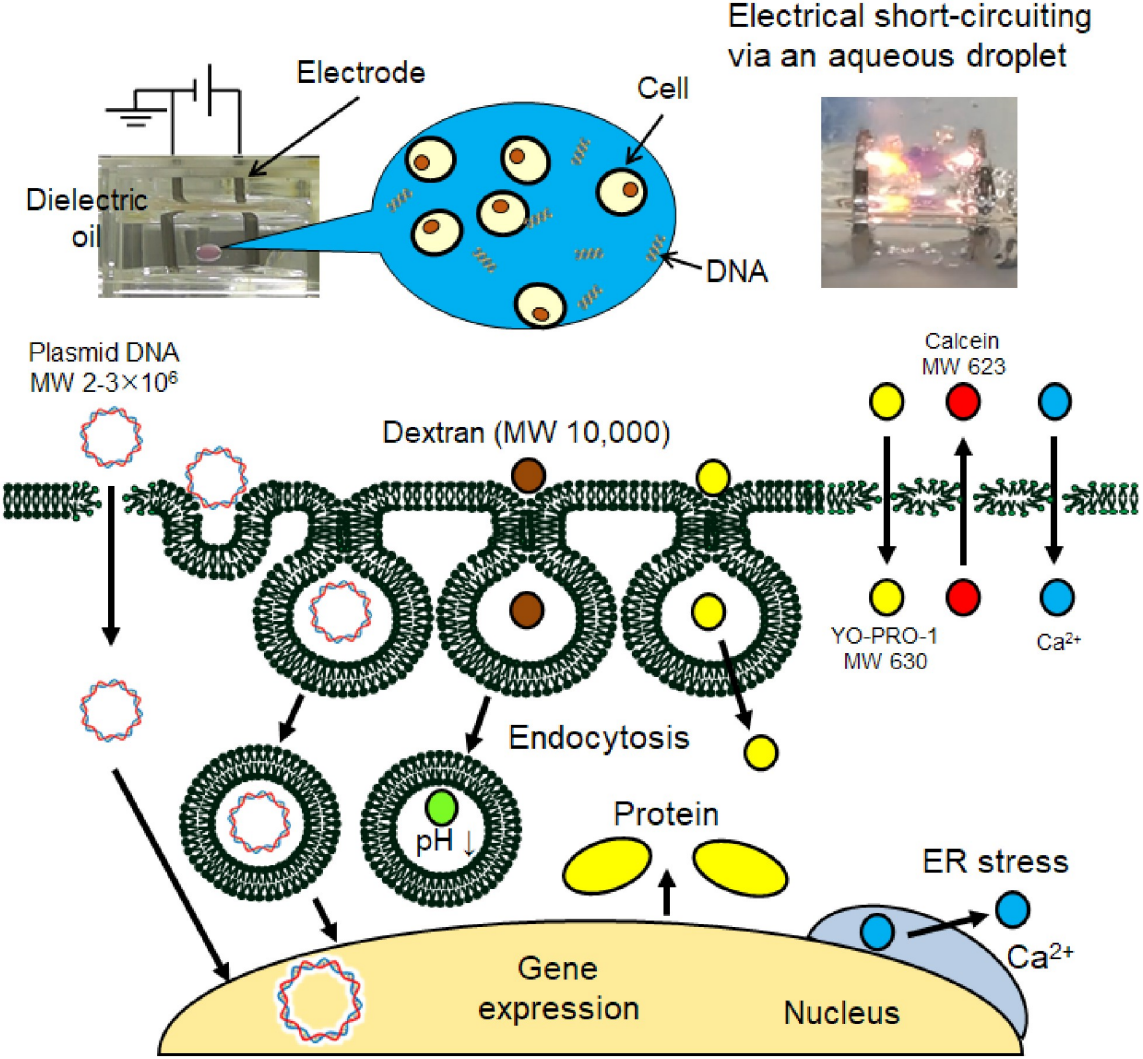
Typical short-circuiting via an aqueous droplet by applying a DC high-voltage electric field and the possible mechanism for gene transfection of mammalian cells investigated in our previous studies.

To date, numerous types of electroporation devices and high-voltage applications have been developed [12]. Our previous studies have demonstrated gene electrotransfer using electrical short-circuiting via a cell suspension droplet in dielectric oil [13, 14]; this method is termed “droplet electroporation” in this paper (Fig 1). An aqueous droplet (a few microliters in volume) dispensed between a pair of electrodes can be deformed by an intense DC electric field depending on the electric field intensity. Elongation of the droplet during deformation connects the electrodes, resulting in a short circuit. When a cell suspension droplet with plasmid DNA is used to short the circuit, gene electrotransfection into various mammalian cells can be achieved. For example, we have demonstrated sufficient gene transfection into human embryonic kidney 293 (HEK293) cells, Jurkat cells, human skin fibroblast cells, mouse suprachiasmatic nucleus (SCN) cells, and bovine and swine fibroblast cells [13–15]. A previous investigation also succeeded in the delivery of proteins into animal sperm. Our methodology has the following features over conventional electroporation: (1) the use of an aqueous droplet in dielectric oil can reduce the number of exogenous molecules and cells. (2) The droplet system can be miniaturized and applied to techniques such as on-chip electroporation. (3) Droplet electroporation can be performed using a DC high-voltage power supply; therefore, an electric pulse generator is not needed.

Even though various electroporation systems have been made available, the mechanisms, such as the delivery of extracellular molecules, cell death, and antitumor effects, are complicated [16]. Generally, electric pulses act on the plasma membrane, and pores are formed in the membrane. This process depends on various parameters, such as field intensity, pulse duration, number of pulses, pulse waveform, temperature, electric conductivity of the medium, and cell properties. Upon removing the electric field, the cell membrane returns to an impermeable state. This process is called membrane resealing or pore resealing. The duration of membrane resealing is typically over a range of minutes and depends on the temperature as well as the extent of pore formation. Cell-impermeable small molecules are considered to be delivered via diffusion throughout the lifetime of the pores; in addition, charged small molecules possibly enter the cells via electrophoresis. In contrast to small molecules, electroporation-stimulated endocytosis has gained acceptance as a core pathway in gene electrotransfection.

**Fig 2:**
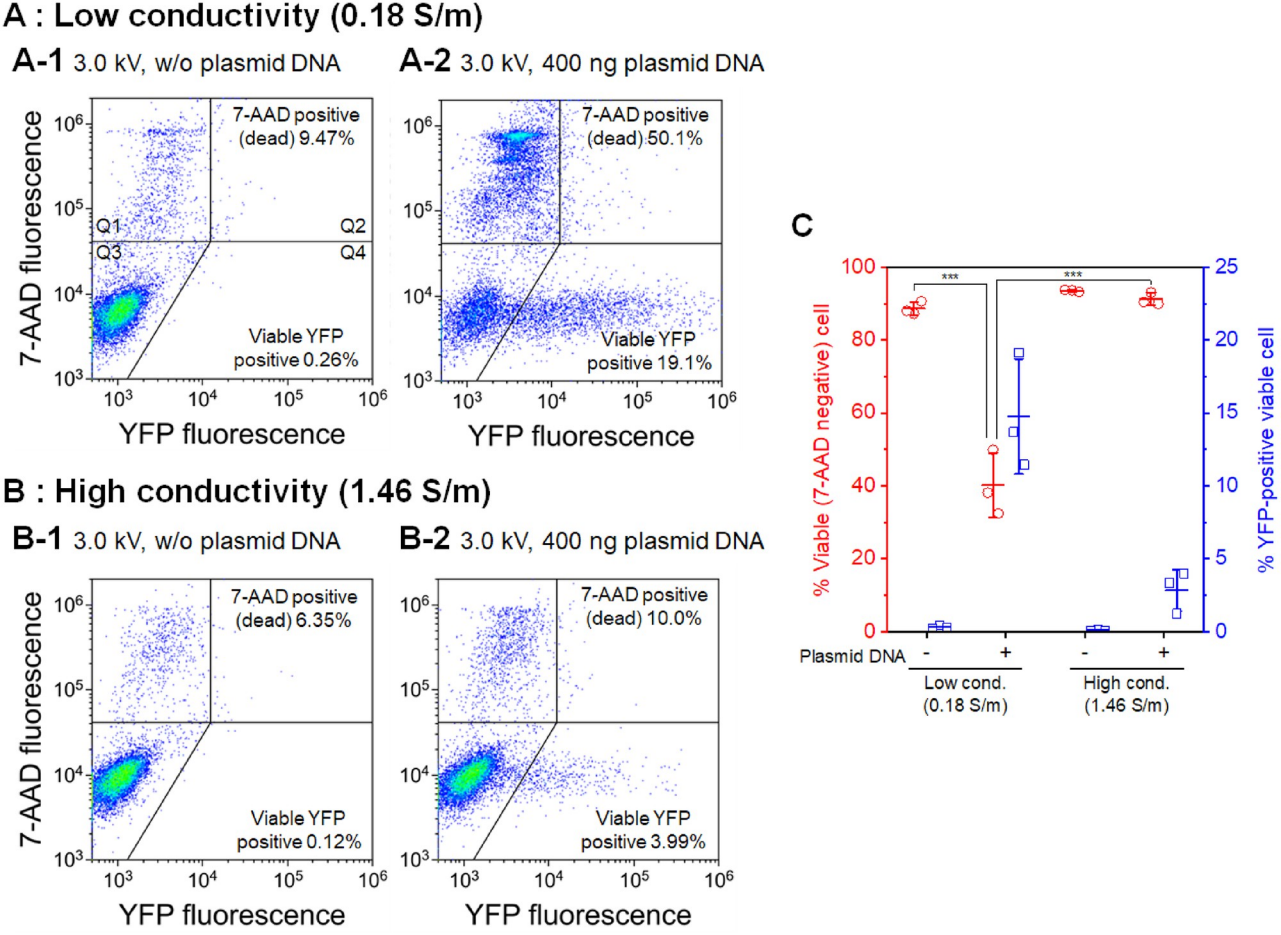
Cell viability and transfection efficiency measured using flow cytometry. Jurkat cells were treated with short-circuiting (3.0 kV, 1 short) via droplets with different electric conductivities and assayed according to 7-AAD uptake for cell death 24 hours after treatment. A, B: The fluorescence intensities of YFP and 7-AAD were measured and plotted using a log scale. The flow cytometry data are displayed as density plots. The results are representative of three independent experiments. The plot has four regions (Q1, Q2, Q3, and Q4). Quadrants Q1 and Q2 include the 7-AAD-positive (dead) cells, Q3 includes the YFP-negative and 7-AAD-negative cells, and Q4 includes the YFP-positive and 7-AAD-negative cells. The percentages of 7-AAD-positive (dead) cells (Q1+Q2) and viable YFP-positive cells (Q4) are shown. C: Cell viability and transfection efficiency 24 hours after short-circuiting, as determined using flow cytometry. Data are expressed as the mean ± standard deviation (SD) of three independent experiments. Statistical significance was determined using one-way ANOVA followed by Tukey’s multiple comparison tests; ***p < 0.001. The raw data are available in the S1 File.

We have also investigated the mechanisms of gene electrotransfection using short-circuiting via an aqueous droplet (Fig 1) [17, 18]. A previous investigation demonstrated that electrical stimulation increases transient membrane permeability to calcium ions and cell-impermeable nucleic acid-binding fluorescent dyes (YO-PRO-1), indicating transient membrane pore formation [17]. Short-circuiting using a low-conductivity electroporation medium enhanced the formation of both transient and irreversible membrane pores. It was also demonstrated that short-circuiting via an aqueous droplet stimulated endocytosis, contributing to successful exogenous gene expression [18]. Taking these investigations into account, this study aimed to investigate the influence of the conductivity of the electroporation medium on gene electrotransfer stimulated by short-circuiting. As a result, a low-conductivity medium with plasmid DNA resulted in a significant decrease in cell viability compared to the high-conductivity medium with plasmid DNA (Fig 2). However, short-circuiting using the low-conductivity medium without plasmid DNA did not show a decrease in cell viability. To assess this phenomenon, we investigated the efflux of small intracellular molecules using calcein-preloaded cells. Calcein acetoxymethyl ester (calcein-AM), a nonfluorescent cell-permeable compound, is hydrolyzed by intracellular esterases, producing calcein (MW = 623), an impermeable fluorescent dye. A decrease in the fluorescence intensity of the cells indicates the leakage of small intracellular molecules [19–22]. Calcein leakage was monitored with different electroporation media, amounts of DNA, structural conformations, and DNA sizes.

## Materials and methods

### Cell culture

All experiments were performed using Jurkat cells (an immortalized line of human acute T-cell lymphocyte cells, RIKEN BioResource Research Center, Tsukuba, Japan). Jurkat cells were maintained in RPMI-1640 with l-glutamine and phenol red (FUJIFILM Wako Pure Chemical, Osaka, Japan), 10% fetal bovine serum (FBS, Thermo Fisher Scientific, Waltham, MA, USA), 100 units/mL penicillin, and 100 *μ*g/mL streptomycin (PS, FUJIFILM Wako Pure Chemical) at 37°C, and 5% CO_2_. Cells were passaged every 2–3 days and a day before the experiment.

### Experimental setup and electrical short-circuiting via a cell suspension droplet in dielectric oil

The droplet electroporation apparatus was fabricated as described in our previous papers [17, 18]. The apparatus consisted of pin headers and a printed circuit board, and the gap between the electrodes was 5.08 mm. The apparatus was filled with fluorocarbon oil (Fluorinert, 3M, Tokyo, Japan), and silicone oil (KF96–100CS, 100 cSt kinematic viscosity, 2.74 relative dielectric constant, 965 kg/m^3^ density Shin-Etsu Chemical, Tokyo, Japan) was added. The cell suspension (3.0 *μ*l) was dispensed in silicone oil, and 3.0 kV of a high voltage was supplied using a DC HV power supply (HJPR-10P6, Matsusada Precision, Shiga, Japan). The maximum output voltage and the current limit were set to 3.0 kV and 0.02 mA, respectively. When the DC HV was applied to the droplet, instantaneous short-circuiting following the droplet deformation could be induced. According to the above setting and the displayed output current during the DC HV application, less than 0.02 mA of the electric current flowed between the electrodes. After short-circuiting, the DC HV power supply was manually turned off. Following the DC HV application, the droplet was recovered and transferred to an appropriate medium for further experiments. Short-circuiting following the droplet deformation could fluctuate, resulting in different exposure times and electric fields to cells. In the untreated control experiment, the droplet containing cells and plasmid DNA was added to the oil and recovered immediately.

### Electroporation media

HEPES-buffered saline solution (HEPES-saline, 10 mM HEPES, 1 mM MgCl_2_, 140 mM NaCl, pH 7.4) and HEPES-buffered sucrose solution (HEPES-sucrose, 10 mM HEPES, 1 mM MgCl_2_, 280 mM sucrose, pH 7.4) were prepared to investigate the influence of electrical conductivity on droplet electroporation. HEPES-saline was used as the high-conductivity medium, and HEPES buffer mixed with HEPES-saline and HEPES-sucrose at a ratio of 1:9 was used as the low-conductivity medium. The electrical conductivities of the high- and low-conductivity media measured using a conductivity meter (LAQUA, Horiba, Kyoto, Japan) were 1.46 S/m and 0.18 S/m, respectively.

### Gene transfection

Gene transfection was conducted as previously described [18] with some modifications. Plasmid DNA expressing Venus yellow fluorescent protein (YFP) was provided by Prof. A. Miyawaki at RIKEN [23]. The plasmid DNA was amplified and purified as described in a previous paper [18]. The DNA concentration was determined using a UV-vis spectrometer (GeneQuant1300, GE Healthcare, Chicago, IL, USA). A 3.0 *μ*l aliquot of appropriate electroporation medium containing 1.0 × 10^5^ Jurkat cells and 0–400 ng of the plasmid DNA was added to the silicone oil. Following the short-circuiting, the droplet was transferred into a microcentrifuge tube containing RPMI-1640/FBS/PS, and the cell suspension was transferred to a 24-well cell culture plate. The cells were cultured for 24 hours at 37°C and 5% CO_2_, harvested by centrifugation, and resuspended in Dulbecco’s phosphate-buffered saline without magnesium chloride and calcium chloride (D-PBS (-), FUJIFILM Wako Pure Chemical). Following resuspension, 7-amino-actinomycin D (7-AAD, Beckman Coulter, Brea, CA, USA) was added to the cell suspension to stain the dead cells, and the cells were incubated for 20 minutes. YFP expression and cell viability were measured using flow cytometry. Viability was calculated by dividing the number of 7-AAD-negative (viable) cells by the total number of cells. Transfection efficiency was calculated by dividing the number of YFP-positive viable cells by the total number of cells.

### Measurement of calcein leakage

The leakage of small intracellular molecules stimulated by droplet electroporation was monitored using a cell-impermeant fluorescent dye, calcein. This experiment was conducted as previously described [17] with some modifications. Jurkat cells were incubated with 0.5 *μ*M calcein-AM (Dojindo, Kumamoto, Japan) in D-PBS (-) for 30 minutes at 37°C. After incubation, the cells were harvested by centrifugation and resuspended in an appropriate electroporation medium, and they were used immediately in droplet electroporation. A 3.0 *μ*l aliquot containing 1.0 × 10^5^ Jurkat cells and 0–400 ng DNA was added to the silicone oil. In the experiments, three types of DNA molecules were used: the Venus YFP-expressing plasmid DNA, pUC19 (2,686 bp, Nippon Gene, Tokyo, Japan), and *λ*DNA (48,502 bp, Nippon Gene). pUC19 DNA was used in both circular and linear forms. Circular pUC19 DNA was linearized by the restriction enzyme *Bam*HI (TOYOBO, Osaka, Japan) and then purified by gel filtration (Clontech CHROMA SPIN-1000, Takara Bio Inc., Shiga, Japan). Following short-circuiting, the droplet was recovered and transferred to the same electroporation medium. The fluorescence intensity of the cells was measured using flow cytometry immediately after short-circuiting. Then, the same sample was measured again after incubation for 20 minutes at 37°C to investigate the resealing after short-circuiting.

### Flow cytometry

The fluorescence intensity of the cells was measured using a CytoFLEX flow cytometer (Beckman Coulter). CytExpert software (Beckman Coulter) was used for data acquisition. Before flow cytometry, each sample was filtered using a nylon cell strainer with a 35-*μ*m mesh size (Corning, NY, USA) to remove cell debris. Kaluza Analysis 2.1 software (Beckman Coulter) was used for data analysis.

## Results

### Influence of droplet conductivity on gene transfection and calcein leakage

Fig 2 shows YFP expression and cell viability 24 hours after droplet electroporation. Fig 2A and 2B show representative flow cytometry density plots using the low-conductivity medium and the high-conductivity medium, respectively. In the case of the low-conductivity medium, Fig 2A-2 remarkably shows an increase in the number of YFP-positive viable cells relative to the absence of the plasmid DNA (Fig 2A-1); however, the number of 7-AAD-positive (dead) cells were also increased. In contrast, short-circuiting using the high-conductivity medium resulted in a slight increase in the number of YFP-positive viable cells (Fig 2B-2) compared to the absence of plasmid DNA, as shown in Fig 2B-1. Fig 2C shows the population of 7-AAD-negative cells (indicating cell viability) and YFP-positive viable cells (indicating transfection efficiency) 24 hours after short-circuiting, as determined from the density plots. Short-circuiting using the low-conductivity medium with plasmid DNA resulted in a statistically significant decrease in cell viability compared to the absence of plasmid DNA; in addition, successful gene transfection was observed. However, short-circuiting using the high-conductivity medium with plasmid DNA did not decrease cell viability. By comparing the electrical conductivity of the media, the low-conductivity medium with plasmid DNA resulted in a statistically significant decrease in cell viability compared to the high-conductivity medium with plasmid DNA. Therefore, short-circuiting using the low-conductivity medium with plasmid DNA resulted in a remarkable decrease in cell viability.

**Fig 3:**
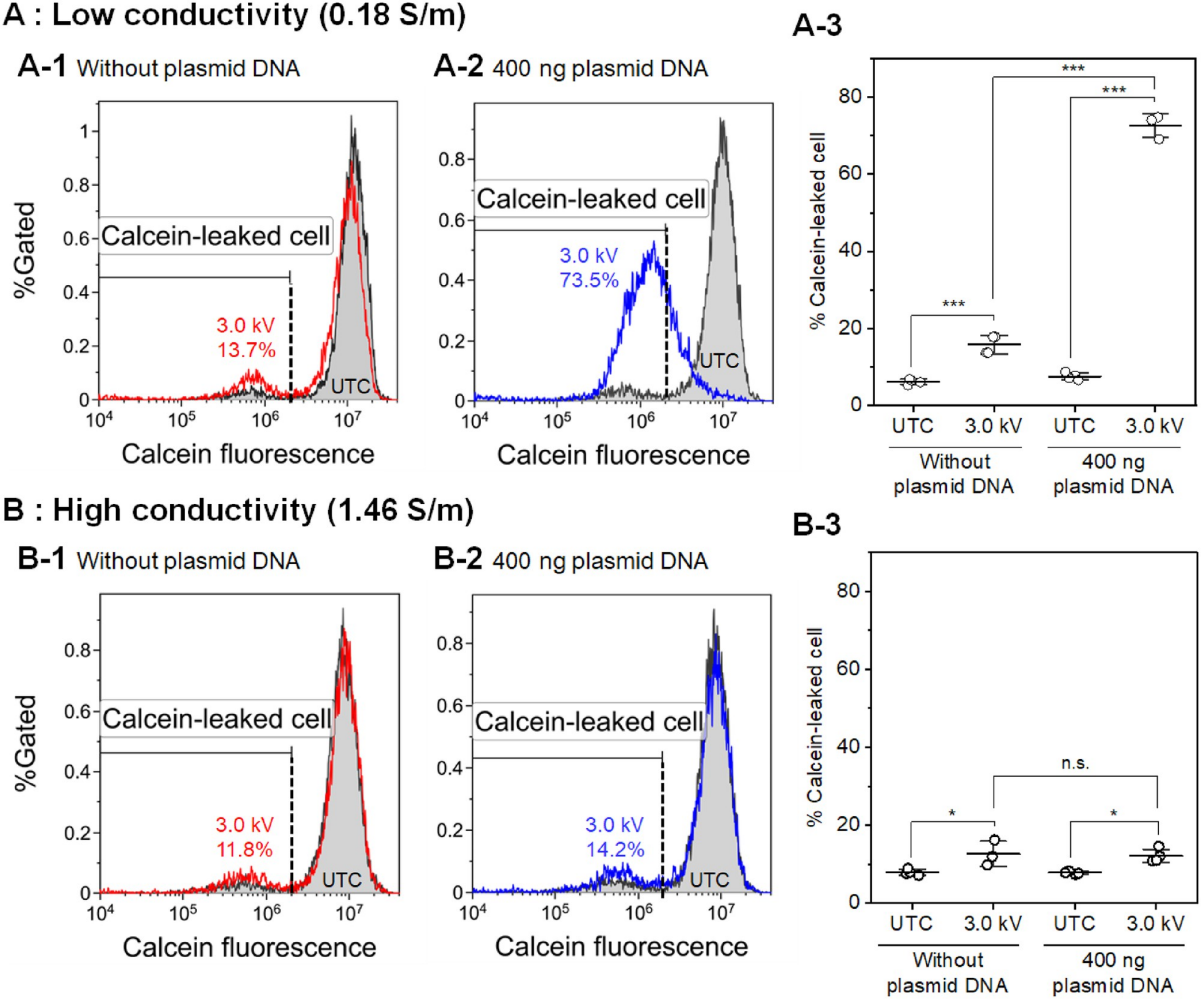
Measurement of calcein leakage stimulated by short-circuiting with and without plasmid DNA. Jurkat cells were treated with short-circuiting (3.0 kV, 1 short) via the droplet with (A) the low-conductivity medium and (B) the high-conductivity medium. (A-1, A-2, B-1, B-2) Typical flow cytometry histograms of calcein fluorescence intensity. The results are representative of three independent experiments. The flow cytometry histogram of the untreated control (UTC) is shown in gray. The black dashed line indicates the threshold of the calcein-leaked cells. The percentage of calcein-leaked cells is shown. (A-3, B-3) The population of calcein-leaked cells, as determined using flow cytometry. Data are expressed as the mean ± SD of of at least three independent experiments. Statistical significance was determined using one-way ANOVA followed by Tukey’s multiple comparison tests; *p < 0.05 and ***p < 0.001. The raw data are available in the S1 File.

In the context of the results of gene transfection using media with different electric conductivities, the efflux of small intracellular molecules was measured using calcein. Fig 3 shows the results of calcein leakage stimulated by short-circuiting using media with different electric conductivities in the presence and absence of YFP-expressing plasmid DNA. Fig 3A shows typical flow cytometry histograms and the population of calcein-leaked cells immediately after short-circuiting using the low-conductivity medium. Short-circuiting using the low-conductivity medium both in the absence and presence of plasmid DNA increased the population of the calcein-leaked cells; in addition, a remarkable increase in the calcein-leaked cells was observed in the presence of plasmid DNA. Fig 3B shows the results of similar experiments using the high-conductivity medium. Short-circuiting using the high-conductivity medium both in the absence and presence of plasmid DNA also increased the population of calcein-leaked cells; however, no statistically significant difference (*p* > 0.05) was observed between the presence and absence of plasmid DNA.

### Influence of the amount of DNA in the low-conductivity droplet on calcein leakage and gene transfection

**Fig 4:**
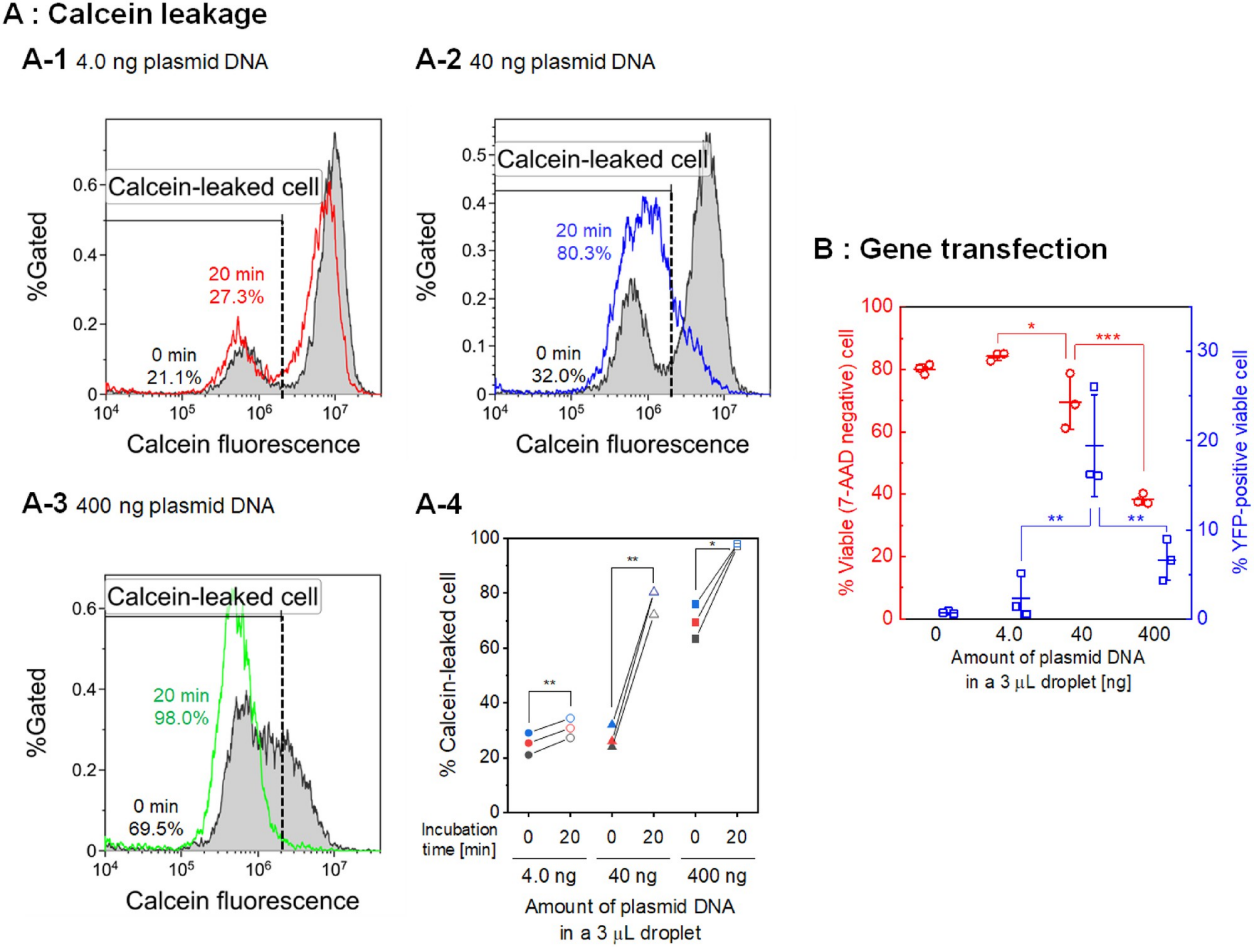
Influence of the amount of DNA in the low-conductivity droplet on calcein leakage and gene transfection. (A) Calcein leakage stimulated by short-circuiting (3.0 kV, 1 short) with a low-conductivity medium containing different amounts of plasmid DNA. (A-1, A-2, A-3) Typical flow cytometry histograms of calcein fluorescence intensity. The results are representative of three independent experiments. The flow cytometry histogram immediately after short-circuiting is shown in gray. The percentage of calcein-leaked cells is shown. The incubation time after short-circuiting is described in minutes. (A-4) The population of calcein-leaked cells, as determined using flow cytometry. Statistical significance was determined using a paired-samples *t* test; *p < 0.05 and **p < 0.01. (B) Cell viability and transfection efficiency 24 hours after short-circuiting, as determined using flow cytometry. Data are expressed as the mean ± SD of three independent experiments. Statistical significance was determined using one-way ANOVA followed by Tukey’s multiple comparison tests; *p < 0.05, **p < 0.01, and ***p < 0.001. The raw data are available in the S1 File.

Figs 2 and 3 demonstrate that short-circuiting using the low-conductivity droplet with plasmid DNA resulted in remarkable calcein leakage and cell death 24 hours after stimulation. Fig 4 shows the influence of the amount of plasmid DNA on calcein leakage and gene transfection after short-circuiting using the low-conductivity medium. Fig 4A shows the results of the calcein leakage experiments with different amounts of YFP-expressing plasmid DNA. In these experiments, the resealing of the cell membrane was also investigated by comparing the calcein leakage immediately after short-circuiting and following incubation for 20 minutes at 37°C. Fig 4A-1–4A-3 show representative flow cytometry histograms. The population of calcein-leaked cells immediately after short-circuiting gradually increased with the amount of plasmid DNA. In the case of 4.0 ng of plasmid DNA, the population of calcein-leaked cells increased by approximately 6% following 20 minutes of incubation after the first flow cytometry measurement. In the case of 40 ng and 400 ng of plasmid DNA, however, the incubation after the first flow cytometry measurement following short-circuiting resulted in a remarkable increase in the population of the calcein-leaked cells. Notably, almost all cells were calcein-leaked 20 minutes after short-circuiting when 400 ng of plasmid DNA was used. Fig 4A-4 shows the population of calcein-leaked cells with different amounts of plasmid DNA and durations of incubation after short-circuiting. A larger amount of DNA shows more significant increases in the population of the calcein-leaked cells both immediately and 20 minutes after short-circuiting. Regardless of the amount of plasmid DNA, a statistically significant increase in the population of the calcein-leaked cells was observed after an incubation of 20 minutes following short-circuiting. Fig 4B shows the results of gene transfection with different amounts of plasmid DNA. Cell viability and transfection efficiency were determined 24 hours after droplet electroporation. Cell viability gradually decreased with increasing amounts of plasmid DNA. The highest transfection efficiency was observed when 40 ng of plasmid DNA was used.

### Influence of DNA characteristics on calcein leakage stimulated by short-circuiting using a low-conductivity droplet

**Fig 5:**
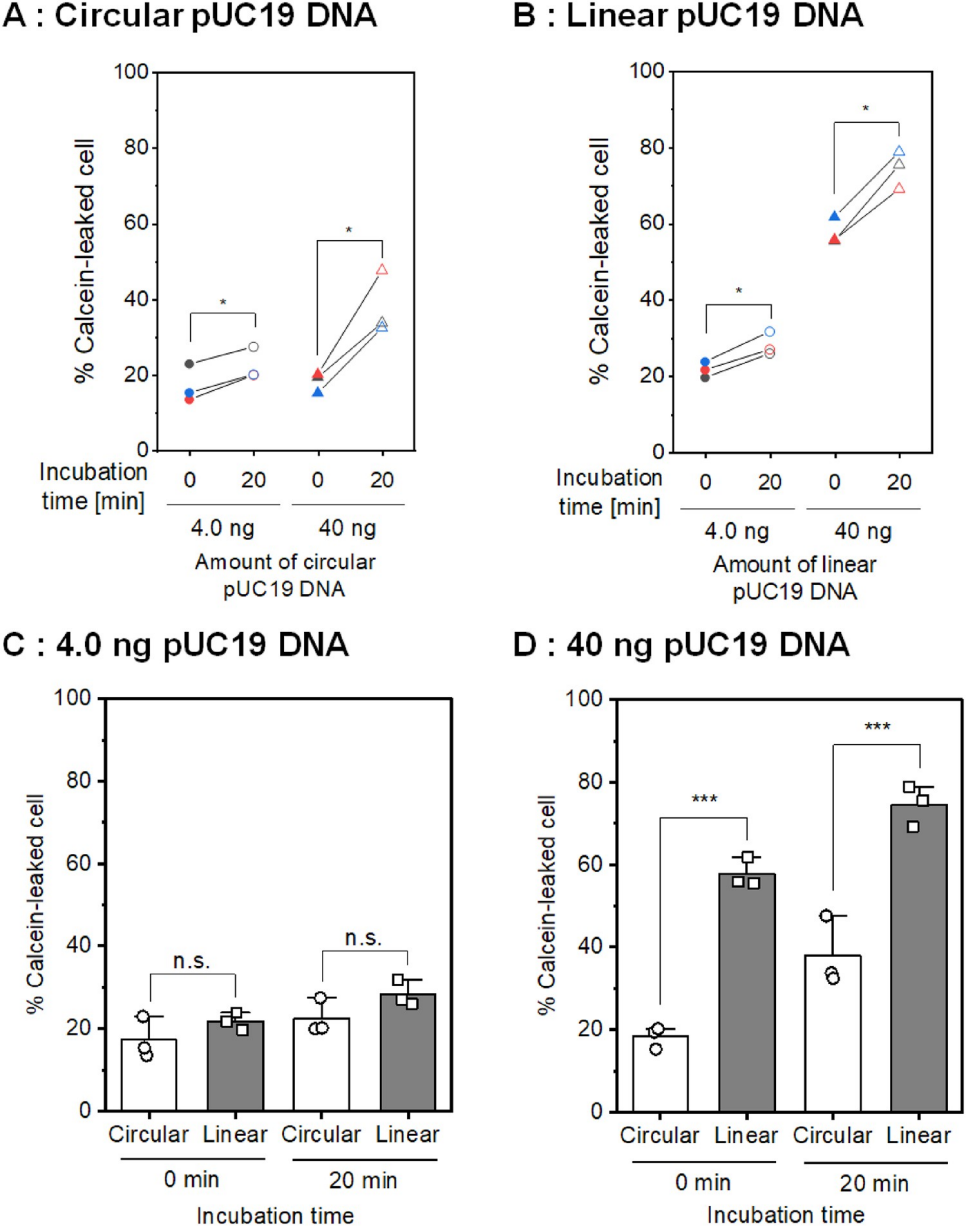
Influence of the structural conformation of plasmid DNA on calcein leakage. The population of calcein-leaked cells stimulated by short-circuiting (3.0 kV, 1 short) using a low-conductivity droplet containing different structural conformations of pUC19 DNA (A: circular, B: linear). Statistical significance was determined using a paired-samples *t* test; *p < 0.05. (C, D) Comparison of the population of calcein-leaked cells between circular and linear pUC19 DNA. The amount of DNA was 4.0 ng (C) and 40 ng (D). Data are expressed as the mean ± SD of three independent experiments. Statistical significance was determined using one-way ANOVA followed by Tukey’s multiple comparison tests; ***p < 0.001. The raw data are available in the S1 File.

Fig 5 shows the results of the calcein leakage experiments investigating the influence of the structural conformation of plasmid DNA in the low-conductivity droplet. Fig 5A shows the population of calcein-leaked cells using 4.0 ng and 40 ng of circular pUC19 DNA. A statistically significant increase in the population of calcein-leaked cells was observed after incubation for 20 minutes following short-circuiting. Fig 5B shows the result of a similar experiment using linear pUC19 DNA. The increase in the population of calcein-leaked cells was also observed 20 minutes after short-circuiting. By comparing the amount of linear pUC19 DNA, 40 ng of linear pUC19 DNA resulted in a higher population of calcein-leaked cells immediately after short-circuiting than 4.0 ng linear pUC19 DNA. Fig 5C and 5D show the influence of the structural conformation of pUC19 DNA on calcein leakage. In the case of 4.0 ng of pUC19 DNA, there was no significant difference (*p* > 0.05) in the population of calcein-leaked cells between circular and linear pUC19 DNA. In the case of 40 ng of pUC19 DNA, however, linear pUC19 DNA resulted in a higher population of calcein-leaked cells than circular pUC19 DNA, both immediately after short-circuiting and after 20 minutes of incubation.

**Fig 6:**
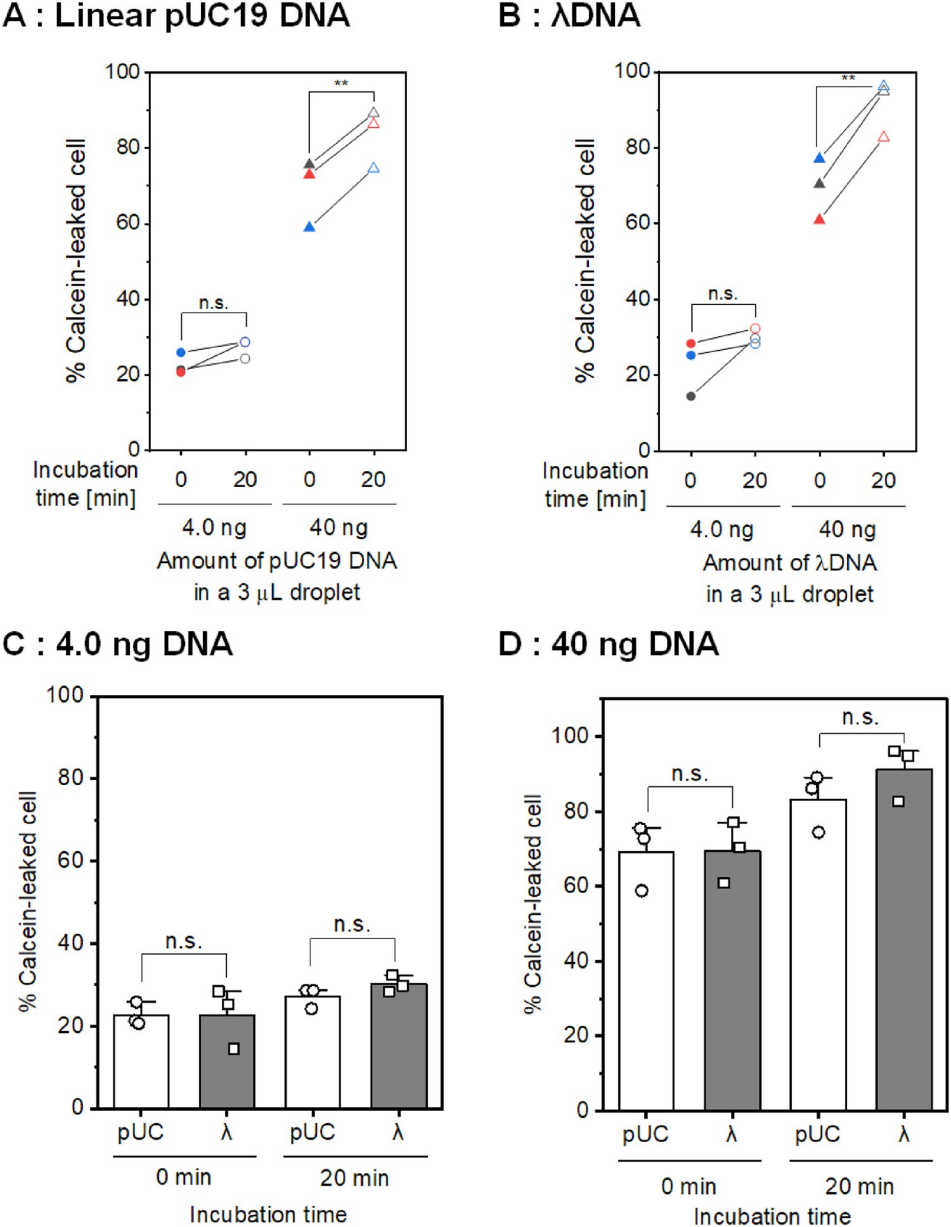
Influence of DNA size in the low-conductivity droplet on calcein leakage stimulated by short-circuiting. The population of calcein-leaked cells stimulated by short-circuiting using a low-conductivity droplet containing different amounts of (A) linear pUC19 DNA and (B) *λ*DNA. Statistical significance was determined using a paired-samples *t* test; **p < 0.01. (C, D) Comparison of the population of calcein-leaked cells between pUC19 DNA and *λ*DNA. The amount of DNA was 4.0 ng (C) and 40 ng (D). Data are expressed as the mean ± SD of three independent experiments. Statistical significance was determined using one-way ANOVA followed by Tukey’s multiple comparison tests. The raw data are available in the S1 File.

Fig 6 shows the results of the calcein leakage experiments with different sizes of linear DNA. Fig 6A and 6B show the population of calcein-leaked cells with different amounts of DNA and time durations after short-circuiting using linearized pUC19 DNA (2,686 bp) and *λ*DNA (48,502 bp), respectively. Regardless of DNA size, a larger amount of DNA resulted in a higher population of calcein-leaked cells immediately after short-circuiting. In addition, the population was significantly increased 20 minutes after the first flow cytometry measurement when 40 ng of pUC19 DNA and *λ*DNA were used; however, no significant increase (*p* > 0.05) in the population of the calcein-leaked cells was observed in the case of 4.0 ng DNA. Fig 6C and 6D show the influence of DNA size on the population of calcein-leaked cells. Regardless of the amount of linear DNA, there was no significant difference (*p* > 0.05) in the population of calcein-leaked cells between linear pUC19 DNA and *λ*DNA.

## Discussion

The aim of this study was to investigate the influence of the conductivity of electroporation medium on gene electrotransfer stimulated by short-circuiting. The low-conductivity medium with plasmid DNA resulted in a significant decrease in cell viability compared to the high-conductivity medium with plasmid DNA. Therefore, we assumed that droplet electroporation using a low-conductivity medium and plasmid DNA would affect membrane damage. As a result of calcein leakage measurement, we demonstrated the influence of DNA characteristics on membrane damage.

Fig 2 shows the influence of droplet conductivity on gene electrotransfer. The gene transfection succeeded with both high- and low-conductivity media, and higher transfection efficiency was obtained using the low-conductivity electroporation medium. However, a significant decrease in cell viability was observed when low-conductivity medium and plasmid DNA were used. The trade-off between cell viability and transfection efficiency could be due to the extent of membrane pore formation. Interestingly, with the low-conductivity medium, we noticed a remarkable decrease in the viability of cells in the presence of plasmid DNA compared to cells in the absence of plasmid DNA. Therefore, we assumed that the presence of plasmid DNA during droplet electroporation using the low-conductivity medium affected the cell membrane damage.

In this study, we analyzed the membrane permeabilization and resealing stimulated by short-circuiting using calcein leakage. Various analyses have been used to investigate membrane permeabilization [4, 20]. The uptake of cell-impermeable nucleic acid-binding fluorophores, such as propidium iodide and YO-PRO-1 iodide, is the most standard way to investigate membrane permeabilization. When these fluorophores enter the cells, they bind to nucleic acids and show a significant fluorescence increase. However, since these fluorophores bind to nucleic acids outside cells, it is difficult to assess pore formation and resealing of the cell membrane stimulated by droplet electroporation with DNA. The influx of calcium ions can also indicate pore formation because calcium ions are present in the cytoplasm at low concentrations. When the cells preloaded with a fluorescent calcium ion indicator conjugated with a cell-permeable acetoxymethyl (AM) ester form are electroporated with calcium ions, a fluorescence increase can be observed using a fluorescence microscope or flow cytometry. This method of analysis can be used to detect small pore formation; however, divalent cations possibly interact with exogenous DNA molecules. For the above reasons, calcein leakage is the most reliable method to assess membrane permeabilization and resealing stimulated by short-circuiting with plasmid DNA.

Fig 3 shows the results of calcein leakage stimulated by short-circuiting with and without 400 ng of plasmid DNA. In the case of the high-conductivity medium (Fig 3B), short-circuiting stimulated a slight increase in the number of calcein-leaked cells compared to the untreated control in both the presence and absence of plasmid DNA. This result suggests transient membrane pore formation resulting in the loss of small intracellular molecules. In addition, plasmid DNA did not enhance calcein leakage compared to the absence of DNA. On the other hand, the population of calcein-leaked cells in the low-conductivity medium with 400 ng of plasmid DNA markedly increased (Fig 3A). These results suggest that plasmid DNA surrounding cells enhanced pore formation using the low-conductivity medium. Our previous investigation demonstrated that short-circuiting using a low-conductivity electroporation medium enhanced the formation of both transient and irreversible membrane pores [17]. As shown in Fig 3A and 3B, however, there was no significant difference in the population of calcein-leaked cells when plasmid DNA was not used. Therefore, as shown in Fig 3, the remarkable increase in the calcein-leaked cells could be attributed to plasmid DNA rather than droplet conductivity.

The increased calcein leakage shown in Fig 3 agrees with the decrease in cell viability shown in Fig 2; therefore, short-circuiting using the low-conductivity medium with a large amount of plasmid DNA could lead to lethal pore formation and subsequent cell death. The mechanisms of cell death induced by electroporation have been discussed in a previous review [24]. The loss of small intracellular molecules such as ATP or ions is one of the possible mechanisms of cell death. Actually, ATP depletion was one of the first investigations to demonstrate membrane permeabilization [25]. Rajeckaitė et al. reported the trade-off between the efficiency of calcein extraction and cell viability, and they considered ATP depletion as the possible cell death mechanism [19]. Jakstys et al. also reported a correlation between the loss of intracellular molecules and cell viability; however, they concluded that ATP depletion did not directly affect cell viability [26]. The calcein leakage investigated in this study could highly correlate with cell death, but further investigation is required to determine the cell death mechanism.

Fig 4A shows the influence of the amount of plasmid DNA on calcein leakage stimulated by short-circuiting using the low-conductivity medium. Here, we compared the fluorescence intensity of the cells immediately after short-circuiting and following incubation for 20 minutes to investigate the resealing of the cell membrane. As shown in Fig 4A, comparing the conditions with 4.0 ng and 40 ng of plasmid DNA, the population of the calcein-leaked cells was not changed immediately after short-circuiting; however, there was a marked increase in the population of the calcein-leaked cells after short-circuiting using 400 ng of plasmid DNA. This result suggests that a more significant amount of plasmid DNA enhanced the membrane pore formation stimulated by short-circuiting using the low-conductivity medium. In addition, there was a statistically significant increase in the population of the calcein-leaked cells with a 20-minute incubation after short-circuiting, regardless of the amount of plasmid DNA. A notable increase in the population of calcein-leaked cells was observed when 40 ng of plasmid DNA was used. In the case of 400 ng of plasmid DNA, the increase in the population of the calcein-leaked cells appears small, but more than 95% of the cells were calcein-leaked cells. Therefore, the results indicate that plasmid DNA enhanced cell membrane damage stimulated by short-circuiting with the low-conductivity medium, and the efflux of small intracellular molecules continued after the short-circuiting.

In Fig 4B, cell viability gradually decreased with the increase in the amount of plasmid DNA. The highest transfection efficiency was observed with 40 ng of plasmid DNA. This result is also attributed to the extent of membrane damage, as shown in Fig 2. The reason that the condition with 400 ng of plasmid DNA did not show the highest transfection efficiency could be due to high cytotoxicity.

Fig 5 demonstrates the influence of the structural conformation of plasmid DNA on calcein leakage. Fig 5A and 5B show the population of calcein-leaked cells using circular and linear pUC19 DNA immediately after short-circuiting and 20 minutes later, respectively. As shown in Fig 5A, the amount of circular DNA did not influence the calcein leakage immediately after the short-circuiting. However, a larger amount of circular DNA inhibited the resealing of the cell membrane. In the case of linear pUC19 DNA, short-circuiting with a larger amount of DNA stimulated a higher efflux of small intracellular molecules. Fig 5C shows no significant difference in the population of calcein-leaked cells between 4.0 ng of circular and linear DNA. However, 40 ng of linear pUC19 DNA resulted in a significant increase in the calcein-leaked cells compared to circular DNA, as shown in Fig 5D. This result suggests that short-circuiting using linearized plasmid DNA stimulated more significant membrane pore formation than circular DNA.

Fig 6 shows calcein leakage stimulated by short-circuiting using different sizes of linear DNA molecules. In this study, we compared calcein leakage with linear pUC19 DNA (2,686 bp; Fig 5) and *λ*DNA (48,502 bp). Regardless of DNA size, conditions with a larger amount of DNA showed a higher population of calcein-leaked cells immediately and 20 minutes after short-circuiting. However, there was no significant difference in calcein leakage between linear pUC19 DNA and lambda DNA. Therefore, the size of linear DNA did not influence calcein leakage.

The effect of DNA in the electroporation medium on membrane damage has been demonstrated in previous studies. In the early 1990s, Klenchin et al. reported increased small molecule delivery that depended on the amount of DNA added [27, 28]. They also compared the membrane permeability with circular plasmid DNA (7.2 kbp) and *λ*DNA, concluding that larger DNA increased the delivery of small molecules. As shown in Figs 5 and 6, on the other hand, linear DNA increased calcein leakage compared to circular DNA, but larger DNA did not. Although the stimulus condition is different, the result of the previous study could be attributed to the difference in the structural conformation, not DNA size. Recently, the effect of extracellular DNA on calcein leakage has been investigated. For example, Ruzgys et al. investigated the improvement of drug delivery using electroporation by adding extracellular DNA [29]. They also showed increased calcein extraction with increasing plasmid DNA concentration. The electric conductivity of their medium was 0.1 S/m; therefore, our result is consistent with theirs. Wang et al. also performed calcein leakage experiments to monitor the extent of membrane damage. They improved cell viability by supplementing either type B gelatin or bovine serum albumin [30]. They also indicated that gene electrotransfer caused more membrane damage than electroporation alone, and the additional damage was mediated by the interactions between plasmid DNA and the cell membrane. However, these studies did not mention the influence of the structural conformation and size of DNA on calcein leakage.

We demonstrated the influence of exogenous DNA on the efflux of small intracellular molecules stimulated by droplet electroporation using a low-conductivity medium. In the context of the previous studies mentioned above, the main findings of this study are as follows: (1) stimulation in conditions with plasmid DNA and low-conductivity medium resulted in tremendous membrane damage; (2) linearized plasmid DNA led to more significant membrane damage than circular DNA; and (3) the size of linear DNA did not influence calcein leakage. Significant membrane damage stimulated by the combination of plasmid DNA and the low-conductivity medium could be attributed to the weakness of the interaction between DNA molecules and counterions in the medium. DNA is a highly charged polymer and its properties depend on the surrounding electrolyte. In this study, the high-conductivity medium contained 140 mM NaCl; therefore, a neutralization effect could influence its contact with a typically negative cell membrane. Negative charges on DNA possibly affect its dynamics in a solution; however, how the structural conformation affects the membrane damage stimulated by electroporation is difficult to prove. The size effect of gene electrotransfer has been previously investigated. For example, Hornstein et al. reported that the entry of circular DNA into cells is not affected by the DNA length; however, vector length contributes to transfection efficiency. They concluded that each step after cell entry, such as endosomal escape, nuclear localization, and transcription, determines the final transfection efficiency [31]. Sachdev et al. investigated the influence of DNA size on DNA-membrane complex formation [32]. They reported that DNA aggregate formation was observed not only for large DNA molecules of 1000 bp but also for short DNA molecules less than 100 bp. Recently, Boye et al. reported that reducing plasmid DNA backbone length significantly increased transgene expression levels, and this effect was observed in both electroporation and a commercially available transfection reagent [33]. Although further investigations are required to prove the detailed mechanism observed here, this study may have implications for understanding the transportation mechanism of electroporation.

## Supporting information

S1 File Raw data table for each figure. (XLSX)
